# Tissue Cytometry Assay with Nuclear Segmentation for Quantifying NETotic Cells in Neutrophils Stimulated by Spermatozoa in Veterinary Species

**DOI:** 10.3390/ani15182742

**Published:** 2025-09-19

**Authors:** Rodrigo Rivera-Concha, Marion León, Nikol Ponce-Rojas, Aurora Prado-Sanhueza, Pamela Uribe, Anja Taubert, Carlos Hermosilla, Raúl Sánchez, Fabiola Zambrano

**Affiliations:** 1 Center of Excellence in Translational Medicine—Scientific and Technological Bioresource Nucleus (CEMT—BIOREN), Edificio Biociencias de la Salud, Faculty of Medicine, Universidad de La Frontera, Avenida Alemania 0458, Temuco 4810296, Chile; rodrigo.rivera@ufrontera.cl (R.R.-C.); a.prado01@ufromail.cl (A.P.-S.); pamela.uribe@ufrontera.cl (P.U.); raul.sanchez@ufrontera.cl (R.S.); 2 Medical Sciences Research Laboratory, Ph.D. Program in Medical Sciences, Faculty of Medicine, Universidad de La Frontera, Montevideo 870, Temuco 4811322, Chile; 3 Ph.D Program in Applied Molecular Cell Biology, Universidad de La Frontera, Avenida Francisco Salazar 01145, Temuco 4811230, Chile; m.leon06@ufromail.cl; 4 Center of Excellence in Morphological and Surgical Studies (CEMyQ), Faculty of Medicine, Universidad de La Frontera, Montevideo 870, Temuco 4811322, Chile; nikol.ponce@ufrontera.cl; 5 Ph.D. Program in Morphological Sciences, Faculty of Medicine, Universidad de La Frontera, Montevideo 870, Temuco 4811322, Chile; 6 Department of Internal Medicine, Faculty of Medicine, Universidad de La Frontera, Claro Solar 115, Temuco 4781218, Chile; 7 Institute of Parasitology, Justus Liebig University Giessen, 35392 Giessen, Germany; anja.taubert@vetmed.uni-giessen.de (A.T.); carlos.r.hermosilla@vetmed.uni-giessen.de (C.H.); 8 Department of Preclinical Sciences, Faculty of Medicine, Universidad de La Frontera, Claro Solar 115, Temuco 4781218, Chile

**Keywords:** neutrophil extracellular traps, spermatozoa, bovine, canine, image cytometry

## Abstract

Upon activation, neutrophils perform three distinct functions: phagocytosis, degranulation of antimicrobial substances into the extracellular medium, and release of neutrophil extracellular traps. Determination of the nuclear area expansion of neutrophils activated to release neutrophil extracellular traps has become critical in demonstrating early neutrophil activation and has become standard. Here, we demonstrate an automated method for measuring nuclear area expansion in two different mammalian species: canine and bovine. For both species, neutrophils were isolated from peripheral blood and co-incubated with fresh spermatozoa for up to 120 min for canine neutrophil–spermatozoa and recently thawed cryopreserved spermatozoa up to 240 min for bovine neutrophil–spermatozoa. Fluorescence images were acquired using a TissueFAXS microscope and then analyzed using StrataQuest v.7.0 software. The images show the release of neutrophil extracellular traps upon activation with spermatozoa for both species, as evidenced by the co-localization of neutrophil elastase and DNA staining. Neutrophil nuclei were expanded as early as 15 min and were detected at up to 120 min in both species. Analysis by nuclei segmentation showed that the data sets generated for both species were reliable and consistent with previously published methods. The method was developed as an automated alternative for measuring the area expansion of neutrophil nuclei in different species.

## Simple Summary

When activated, neutrophils do three things: phagocytize, liberate chemicals that battle infection, and release traps that catch and kill pathogens. Determining the nuclear area expansion of activated neutrophils is critical for demonstrating early neutrophil activation and has become standard. Here, we show an automated method for measuring how much the nucleus of neutrophils expands in two different mammals: dogs and cows. For both species, neutrophils were separated from the blood and incubated with fresh sperm. For dog samples, the neutrophils and sperm were incubated for 120 min, and for cow samples, the neutrophils and sperm were incubated for 240 min. Fluorescence images were taken using a fluorescence microscope and then analyzed. The images show the release of neutrophil extracellular traps when they are activated by spermatozoa for both species. This is shown by the neutrophil elastase and DNA staining being in the same place. The nuclei of cells expanded by as early as 15 min and were detected up to 120 min in both species. The analysis showed that data sets from both species were reliable and consistent with other methods that had been published. This method was created to measure neutrophil nuclei expansion in different species automatically.

## 1. Introduction

Polymorphonuclear neutrophils (PMN) are the most abundant leukocytes in humans, canines, and other mammals, comprising approximately 50–70% of the bloodstream [1]. These cells are the first to arrive at sites of infection or inflammation. A distinguishing feature of PMN is the presence of a multilobulated nucleus [2], which is the etymological origin of the term. In situ, PMN perform three primary functions: First, phagocytosis is defined as the internalization of pathogens by PMN via endocytosis, leading to their subsequent destruction. Second, degranulation involves the release of molecules and proteins with antimicrobial activity. Finally, the release of neutrophil extracellular traps (NETs) is a critical component of the immune response [3,4]. As previously documented, the primary components of NETs are DNA and histones that bind to proteins. It has been determined that 70% of all proteins are derived from NETs. Several proteins have been identified that play a role in the process of NET formation. These include antimicrobial proteins from PMN granules such as myeloperoxidase (MPO) [5], neutrophil elastase (NE) [6], and others [7]. As previously documented, NETs have been shown to trap and destroy pathogens, including bacteria [3,8], fungi [9,10], and parasites [11,12,13].

There are two mechanisms of NET formation: The first one involves the death of PMN, through a process called suicidal NETosis. The other—an early one where the NETs are liberated in vesicles to the extracellular medium, and the PMN remains alive—is referred to as vital NETosis [14]. In the case of suicidal NETosis, it is initiated by the activation of PMN followed by the activation of the enzyme NADPH oxidase (NOX) [15,16]. This results in the generation of a peak of reactive oxygen species (ROS). The subsequent translocation of the enzyme peptidyl arginine deiminase type IV (PAD4) citrullinates histones, leading to decondensation of chromatin [17]. This process causes nuclear elongation. This is followed by rupture of the nuclear membrane and disintegration of the cytoplasmic granules, culminating in rupture of the plasma membrane. As a result, all internal contents are shed into the extracellular medium, forming NETs [4]. Although NETs play a critical role in the regulation of pathogens, their exacerbation and inadequate removal have been associated with a variety of inflammatory pathologies. This multifaceted relationship has led to the characterization of NETs as a “double-edged sword” [18].

In bovines, the presence of PMN in the female reproductive tract (FRT) has been associated with impaired fertility in artificial insemination [19] and decreased fertility [20]. In canines, the NETs’ release has been described as being activated by the parasite *Angiostrongylus Vasorum* in vitro [21]. Our group was the first to characterize the NETs’ release as activated by canine spermatozoa, and to record the adverse effects of NETs’ components on spermatozoa [22].

It is well established that one of the initial stages of PMN activation is chromatin decondensation and nuclear expansion, resulting in an increase in nuclear and cell volume [23]. Consequently, the measurement of nuclear area expansion (NAE), also referred to as the NETotic process, has become a common practice in 2D microscopic imaging. The first documented case of this method occurred in 2010 [5,6], and it was subsequently standardized in 2014 [24]. The procedure involves manual identification of PMN nuclei using ImageJ software and subsequent determination of the area of each selected nucleus. Its use for PMN of different species has been documented over several years [21,25].

The use of microscopy images for quantitative analysis has facilitated the development of numerous software tools that facilitate the recognition of tissues and cells within a specimen preparation. In such cases, the identification of different cells and organelles within an image is advantageous. This identification can be facilitated by examining images of either tissue slides (ex vivo) or cultured cells (in vitro) [26]. This form of identification is referred to as “instance segmentation” [27], although in life sciences it is more commonly referred to as “segmentation” [28,29]. In cases where this identification involves the nucleus, the term “nuclei segmentation” [30] is used. Nuclear segmentation allows the identification and location of nuclei in a tissue sample.

In our laboratory, we have previously used nuclei segmentation to measure NAE in bovine and canine PMN activated by spermatozoa in vitro [22,31]. The purpose of this study is to present a protocol for measuring NAE in canine and bovine PMN activated by spermatozoa, thereby inducing the release of NETs.

## 2. Materials and Methods

### 2.1. Canine Sperm Sample Acquisition

The canine semen samples were obtained from three healthy animals (3 semen samples per dog were used for the experiments). The animals were of three breeds: a 3-year-old Chihuahua, a 2-year-old Cattle Dog, and a 3.5-year-old Dachshund. The samples were centrifuged at 300× *g* for 5 min. This procedure was implemented with the objective of extracting the seminal plasma.

### 2.2. Bovine Sperm Selection

Bovine sperm cryopreserved in liquid nitrogen were thawed at 37 °C for 1 min. Thereafter, the samples were separated using a Bovipure density gradient kit (Nidacon, Gothenburg, Sweden) with centrifugation at 600× *g* for 5 min. The samples were then subsequently washed twice with 800 μL of Sperm-Talp medium as described by Bavister and Yanagimachi [32], with some modifications [33]; centrifuged at 300× *g* for 4 min; and finally resuspended in 200 μL of Sperm-Talp medium.

### 2.3. Canine PMN Isolation

Blood samples for PMN isolation were obtained from healthy dogs (n = 3) by cephalic vein puncture at the University Veterinary Hospital. Next, 2 mL blood in EDTA tubes was applied to 2 mL Histopaque 1077/Histopaque 1119 (Sigma-Aldrich, St. Louis, MO, USA) density gradient and centrifuged at 340× *g* for 30 min at room temperature (RT) in a U-32R swing-rotor centrifuge (Boeckel GmbH & Co., Hamburg, Germany) without brake. The supernatant containing plasma and peripheral blood mononuclear cells (PBMCs) was removed, and the PMN sediment was carefully isolated by pipette aspiration. The sediment was then washed with a sterile Hank’s balanced salt solution (HBSS) (Biochrom AG, Berlin, Germany), followed by centrifugation at 300× *g* for 10 min, and the resulting pellet was resuspended in sterile lysis buffer and gently mixed for 10 min. The pellet was then washed twice with sterile HBSS medium and the PMN were resuspended in HBSS. Finally, the viability and purity of PMN were analyzed by exclusion with trypan blue (Sigma-Aldrich, St. Louis, MO, USA) in a Countess 3 FL system (Invitrogen, Thermo Fisher Scientific, Waltham, MA, USA).

### 2.4. Bovine PMN Isolation

Bovine PMN were isolated from peripheral blood of dairy cows (n = 4) as described by Roth and Kaeberle [34] with some modifications [35]. Briefly, peripheral blood was collected from the jugular vein using the Vacutainer system in tubes containing EDTA to prevent coagulation. Then, 20 mL of blood was diluted in 20 mL of sterile PBS supplemented with 0.2% EDTA. This dilution was then applied on top of a Hystopaque-1077 Separation Solution and centrifuged at 800× *g* for 45 min. The supernatant was discarded, and the erythrocytes were lysed with 20 mL lysis buffer for 1 min. Tonicity was restored with a hypertonic solution, which was centrifuged at 600× *g* for 10 min; the supernatant was discarded; and the remaining pellet was washed twice with 40 mL of HBSS, centrifuged again at 600× *g* for 10 min, and finally resuspended in 3 mL of HBSS. The viability and purity of isolated bovine PMN were analyzed by exclusion using a commercial Trypan blue test in a Countess 3 FL system (Invitrogen, Carlsbad, CA, USA).

### 2.5. Immunofluorescence of NE

PMN were incubated with bovine spermatozoa at a 1:3 ratio (2.5 × 10^5^ PMN to 7.5 × 10^5^ spermatozoa) for 15 to 240 min at 37 °C in 5% CO_2_. For the canines, the PMN were incubated with canine spermatozoa for 15 to 120 min in the same conditions as described above. They were fixed with 4% *p*-formaldehyde for 15 min, washed with sterile PBS, and blocked with PBS supplemented with 2% bovine serum albumin (BSA) for 30 min at RT. Samples were incubated overnight (15 h) with a rabbit polyclonal anti-NE antibody (Abcam, Cambridge, UK) in PBS—2% BSA at a 1:300 dilution at RT. The cells were washed three times with 200 μL of PBS shaking at 100 rpm for 5 min, then incubated with a goat anti-rabbit polyclonal IgG antibody conjugate with Alexa Fluor 488 (Invitrogen, Carlsbad, CA, USA) in sterile PBS—2% BSA at a 1:500 dilution for 1 h at RT, protected from light. For staining extracellular DNA, samples were incubated with Sytox Orange (Invitrogen, Carlsbad, CA, USA) at 1:2000 dilution in sterile PBS for 15 min at RT, washed with sterile PBS, and mounted on Fluoromount-G mounting medium containing DAPI (Invitrogen, Carlsbad, CA, USA) for subsequent visualization using TissueFAXS i Plus Cytometry (Tissue Gnostics, Vienna, Austria).

### 2.6. TissueFAXS i Plus Cytometry System

The TissueFAXS i Plus Cytometry System consists of a ZEISS Axio Observer 7 brightfield and epifluorescence microscope (Carl Zeiss Microscopy, Jena, Germany) equipped with a TissueFAXS i Plus inverted automated imaging system (Tissue Gnostics, Vienna, Austria). This system contains a Märzhäuser motorized stage and a sCMOS monochromatic camera (16-bit, 2048 × 2048 px) for fluorescence and a CMOS color camera (8-bit, 2048 × 2048 px) for brightfield. The operation of these systems is controlled by TissueFAXS v. 7.0 scanning and management software. The instrument is equipped with LED illumination and Chroma multi-LED set filters (Chroma Technology Corporation, Bellows Falls, VT, USA). For the acquisition of Alexa Fluor 488 and Sytox Orange fluorescence, LED N°3: 475 nm and LED N°5: 555 nm were used for excitation and quadband filters for DAPI/Cy2/Cy3/Cy5 were used for emission. The software allowed us to select the optimal reflector configuration for each fluorophore. In this particular case, the “FITC” reflector selected LED N°3 and Cy2 filter for Alexa Fluor 488, and the “TEXA” reflector selected LED N°5 and Cy3 filter for Sytox Orange.

Image processing was performed by context-based analysis using StrataQuest v.7.0 software (TissueGnostics, Vienna, Austria).

### 2.7. Analysis of NETotic Cells by Nuclear Area Expansion (NAE)

Nuclear segmentation was used to detect NETotic cells by NAE [28,36], an automated method that can define and detect the nuclei in a sample treated with histochemical, immunocytochemical, and/or immunofluorescence techniques. In an epifluorescence-based technique, the fluorescence intensity (FI) threshold is defined to distinguish background noise from the FI of the nucleus [29]. In the case of bovine samples, the fluorescence signal of Sytox Orange was measured over an area of 5 mm^2^, avoiding artificial bubbles. The “Nuclei Mask” layer was used for nuclear segmentation, and the thresholds of 10% smallest size and 10% lowest FI were manually compensated using the layer editor to eliminate the detection of sperm nuclei and cell debris. The “measuring inside ROI” command was used to avoid measuring cells that were only partially included in the region of interest (ROI). All PMN nuclei detected within the 5 mm^2^ area were counted, and the area occupied by each PMN nucleus was determined in μm^2^ using StrataQuest v. 7.0 software (TissueGnostics, Vienna, Austria). A minimum of 2.0 × 10^4^ nuclei were counted for each experimental NAE assay. A PMN was considered NETotic with an NAE greater than 80 μm^2^, as reported by González et al. [24].

For canine PMN, the nuclei average area was considered, and nuclei areas below 14 μm^2^ were filtered using the “Nuclei Mask” layer editor. For each treatment, five 4 mm^2^ quadrants were randomly selected and Sytox Orange fluorescence was measured. This approach allowed us to filter spermatozoa nuclei. PMN nuclear areas were analyzed using StrataQuest v.7.0 software (TissueGnostics, Vienna, Austria).

### 2.8. Ethics Statement

The animal study protocol was approved by the Scientific Ethics Committee of Universidad de La Frontera, Temuco, Chile (authorization code 120_20); and in accordance with Chilean Statute N° 20,380 on “Protection of Animals”.

### 2.9. Statistical Analysis

In case of bovine PMN, independent experiments were performed at least three times with different semen samples from the same bull. In the case of canine PMN, for the immunofluorescence experiments, one smear per sample obtained on different days with an n = 8 was evaluated. The results were presented as mean ± standard deviation (SD). The GraphPad Prism software v. 10.1.1 was used for the statistical analyses. An exploratory analysis of data was conducted using the D’Agostino–Pearson K^2^ and Shapiro–Wilk tests to evaluate the normal distribution. If they failed to pass the normality test, the values were transformed into their arcsine value. Student’s *t*-test was used to evaluate significant differences between each time slot. A level of *p* < 0.05 was considered significant.

## 3. Results

**Figure 1:**
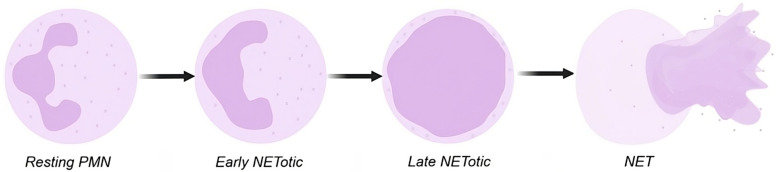
Diagram of nuclear area expansion from a PMN activated to release NETs. Resting PMN: There is no activation. The early NETotic stage involves nuclear decondensation and expansion. The late NETotic stage involves a mixture of nuclear and cytoplasmic components and NET. Finally, a membrane disruption occurs, and the NET is released.

The schematic diagram (Figure 1) shows the nuclear area expansion, perceived in two dimensions, that PMN undergo upon activation, ultimately resulting in the release of NETs. The determination of this expansion can be achieved through the use of nuclei segmentation.

**Figure 2:**
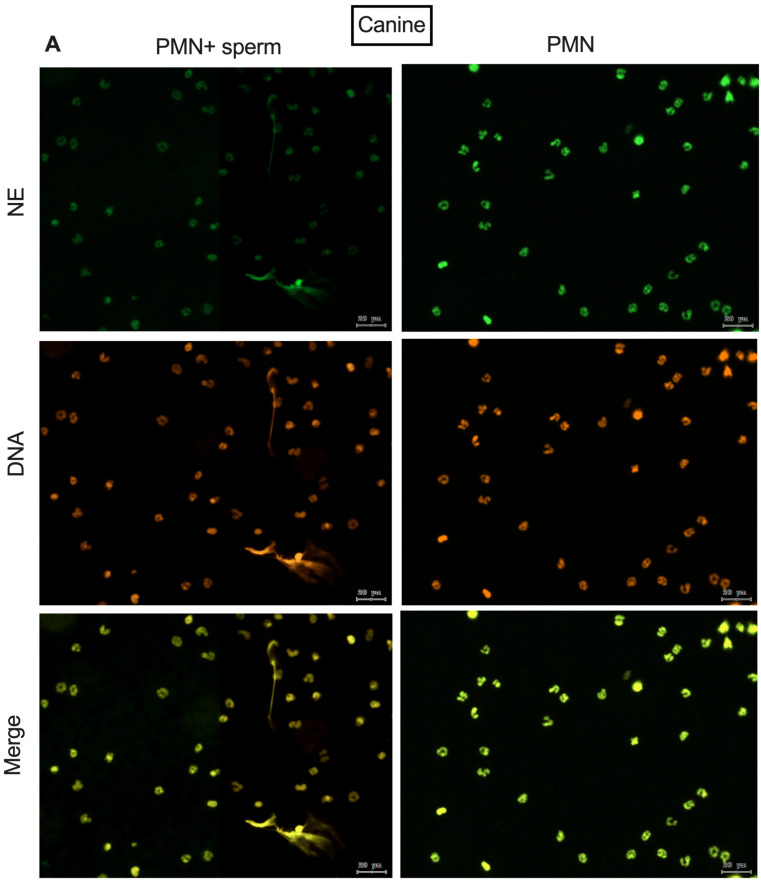

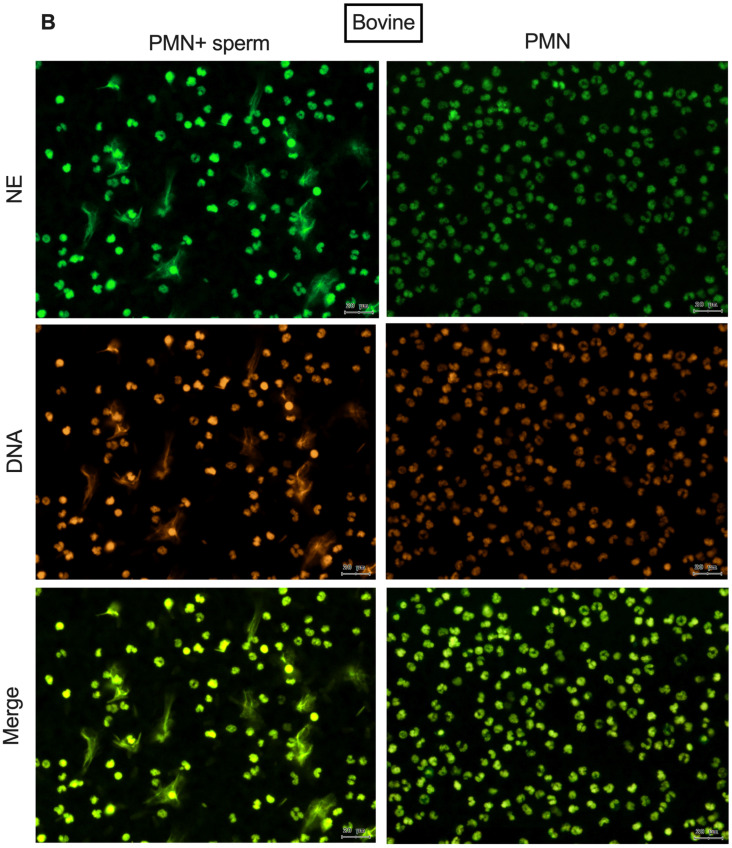
Representative images of immunofluorescence of NE and Sytox Orange DNA staining. All images were taken at 2 h of co-culture as follows: (**A**) canine PMN + viable sperm, (**B**) bovine PMN + viable sperm.

Figure 2 shows the immunofluorescence of NE and the colocalization between NE and DNA after 2 h of co-incubation of PMN. It features spermatozoa for both species, demonstrating the release of NETs activated by spermatozoa as already described in the literature.

**Figure 3:**
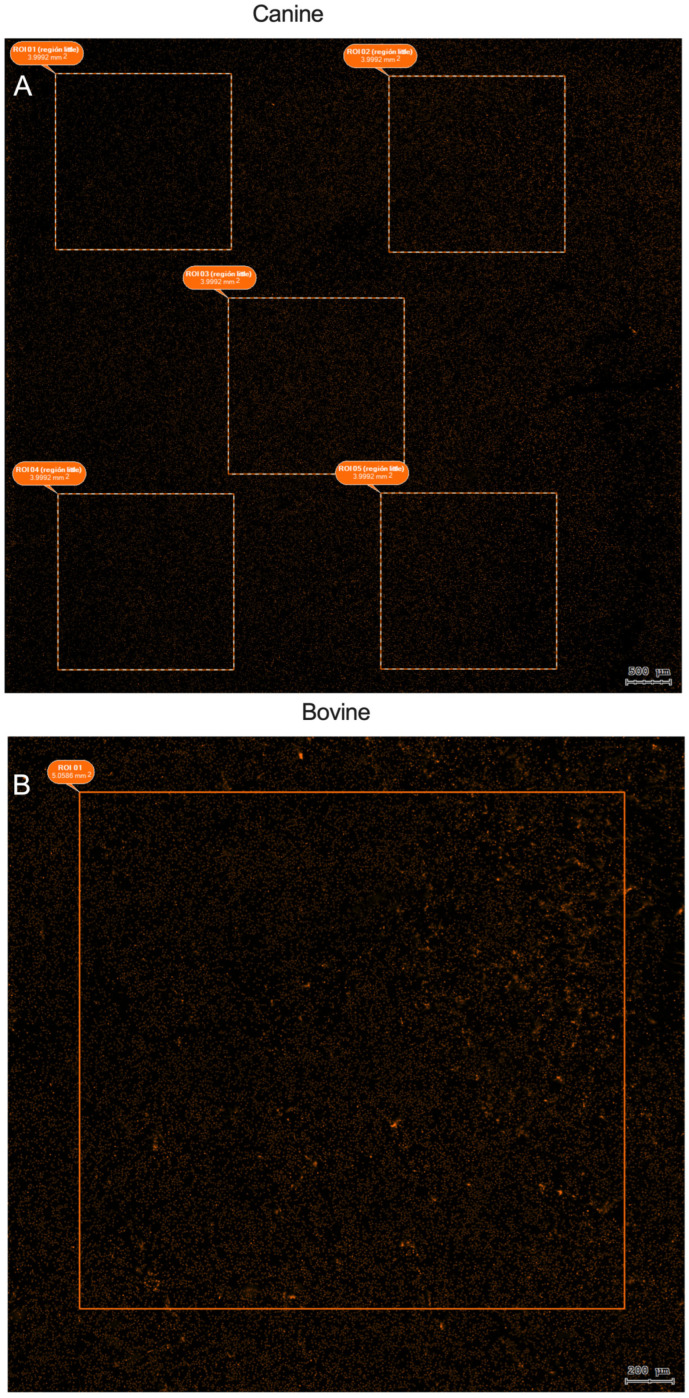
Representative images for the ROIs strategies used to analyze PMN nuclei with Sytox Orange DNA stain in co-culture of (**A**) canine PMN + spermatozoa, and (**B**) bovine PMN + spermatozoa, both at 15 min of co-incubation.

Once the images were acquired and all of the samples were scanned, the StrataQuest software was used for contextual analysis. First, the ROI was selected, and, as shown in Figure 3, different strategies were used to sample the PMN nuclei, and their areas were measured for the different species.

**Figure 4:**
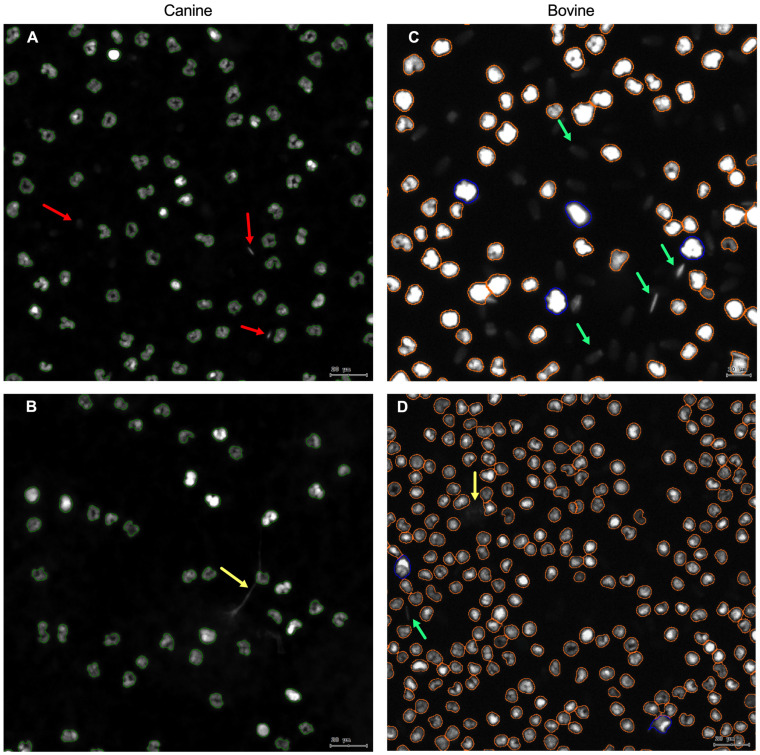
Representative image of the nuclei mask using Sytox Orange DNA stain in co-cultures of (**A**,**B**) canine PMN—spermatozoa, and (**C**,**D**) bovine PMN—spermatozoa. Green arrows: bovine sperm heads; red arrows: canine sperm heads, yellow arrows: different morphotypes of NETs; green surrounding lines: canine PMN nuclei mask; orange surrounding lines: bovine PMN nuclei < 80 μm^2^; blue surrounding lines: bovine PMN nuclei > 80 μm^2^. All images were obtained using a 20× objective.

Figure 4 shows nuclei segmentation using the Sytox Orange DNA stain in a co-culture between bovine PMN and spermatozoa. The StrataQuest software was able to delineate the nucleus of each PMN within the ROI. The “nuclei mask” layer was able to filter by size and fluorescence intensity. Each project was evaluated based on a set of qualitative criteria. This allows the exclusion of sperm heads by size and NETs by size and fluorescence intensity. In the case of canine co-cultures, exclusion was primarily based on size, as most sperm heads had bright fluorescence intensity but a small nucleus size; and in the case of bovine co-cultures, the exclusion was primarily based on fluorescence intensity, as sperm heads had dimmer fluorescence intensity than PMN nuclei.

**Figure 5:**
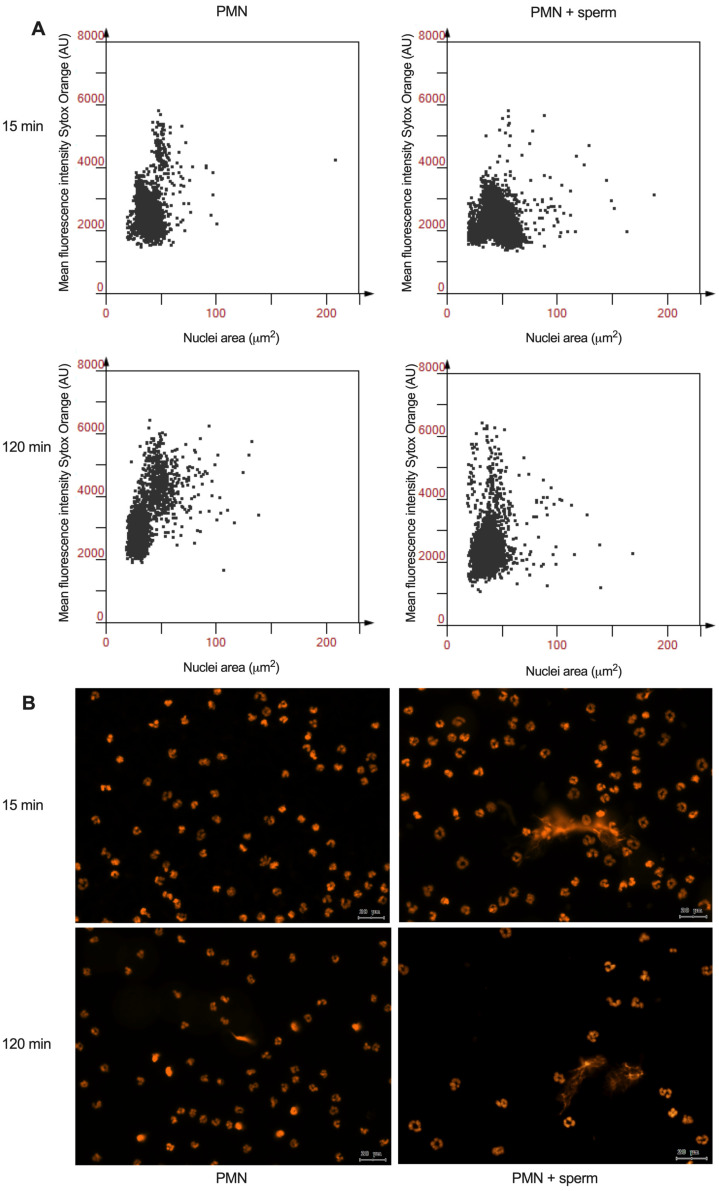
Canine NETotic PMN via nuclei area expansion (NAE) analysis by tissue cytometry in the absence and presence of spermatozoa. (**A**) Representative scattergrams for the nuclei segmentation of PMN, (**B**) representative images for the Sytox Orange DNA stain for the same treatments described above.

The representative scattergrams generated by the analysis from the software StrataQuest for the NAE of canine PMN demonstrate an expansion in the nuclei area for PMN after as little as 15 min of co-incubation. This phenomenon is sustained after 120 min of co-incubation, as shown by comparison with PMN alone. Representative images of the Sytox Orange DNA staining from which the analysis was derived are shown in Figure 5B.

**Figure 6:**
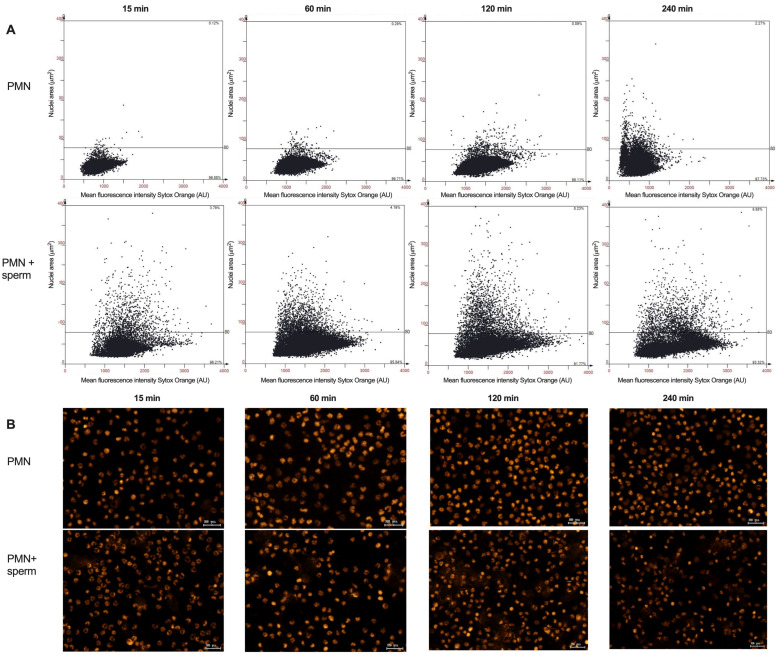
Bovine NETotic PMN via nuclei area expansion (NAE) analysis by tissue cytometry in the absence and presence of spermatozoa. (**A**) Representative scattergrams for the nuclei segmentation of PMN, the threshold for a nucleus to be NETotic is 80 μm^2^. (**B**) Representative images for the Sytox Orange DNA stain for the same treatments described above.

Figure 6A shows representative scattergrams generated by the analysis from the software StrataQuest for the NAE of bovine PMN in co-culture with spermatozoa, showing the threshold of 80 μm^2^, above which it is considered a bovine PMN NETotic. Figure 6B shows representative images for the Sytox Orange DNA staining from which the analysis was derived.

The scattergram-derived quantification of NAE (Table 1) illustrates the different strategies for presenting the results. In the case of canine PMN (Table 1A), no resting neutrophil nuclear area has been reported to date. Therefore, the results are presented as the mean of the PMN’ nuclei area. A significant difference in nuclei area was observed between PMN alone and PMN co-incubated with spermatozoa after 15 min of co-incubation. This difference persisted for up to 120 min. In the case of bovine PMN, there is evidence supporting a resting PMN nucleus area of 80 μm^2^ [24]. Therefore, for this species, results are presented as the percentage of PMN nuclei with an area greater than 80 μm^2^. So, there is a significant difference in the percentage of NETotic PMN nuclei in co-cultured PMN–spermatozoa compared with PMN alone. This difference was evident as early as 15 min after initiation of co-culture and persisted for up to 120 min. This finding demonstrates the role spermatozoa plays in initiating the NETotic process.

## 4. Discussion

Bovine-assisted reproductive techniques (ARTs), particularly artificial insemination, have been associated with lower fertility rates in the presence of subclinical inflammation [37,38]. This phenomenon has been associated with the presence of leukocytes, especially PMN, in the female reproductive tract (FRT) [20]. Fichtner et al. [39] and Fichtner et al. [40] measured NETs’ release in the presence of spermatozoa at 1 h of incubation using spectrofluorimetric assays and scanning electron microscopy; however, this kind of approach does not account for the early activation of PMN. In canines, reproductive failure in bitches associated with infectious diseases of the FRT contributes to a lower fertility rate [41]. The initial stages of PMN activation to release NETs involved activation of NOX [16] and translocation of NE and PAD4 to the nucleus, followed by nuclear decondensation and a subsequent increase in nuclear volume [17].

Attempts have been made to measure nuclear volume increase; however, this process is challenging and time-consuming [23], so it has become standard to measure NAE on a 2D fluorescence image. Papayannopoulos et al. and Metzler et al. [5,6] used ImageJ processing software, with each investigator individually counting 300–500 cells per well. This method was standardized in 2014 [24]. In our laboratory, the same method was used for human vaginal discharge smears, with cell counts ranging from 786 to 1757 cells per smear. On the other hand, Grob et al. [12] and Grob et al. [21] measured NAE on parasite-activated PMN in vitro using ImageJ in conjunction with DNA Area and NETosis Analysis (DANA) software I and II. The number of cells counted ranged from 200 to 300 per well [42].

The main advantage of the present approach is the use of TissueFAXS scanning capabilities, which allow us to record and photograph all fluorescence channels for the entire sample, and the StrataQuest v. 7.0 software; these capabilities use context-based analysis through a nuclei segmentation algorithm that allows us to identify only nuclei in our sample, counting between 1.5 × 10^4^–2.5 × 10^4^ nuclei. The use of this approach ensures the automation of the process, thereby eliminating potential biases that may be introduced by subjective interpretation by researchers unfamiliar with the method.

Semiautomated NET detection using ImageJ software has been described for human PMN [43] and has been used to detect bovine NET release activated by parasites [44]. The method described here does not measure NETs release. Rather, it quantifies the initial stage of PMN activation, specifically the nuclear decondensation that occurs before NET release. The challenges associated with this method primarily stem from two factors. Firstly, there is a communication gap between the researcher and the microscope operator. The researcher understands the intricacies present in their samples, while the operator is proficient in acquiring images suitable for subsequent analysis. Secondly, the method involves the management of voluminous data, as the generated images encompass the entire slide. These challenges are shared with other image analysis methods [45], and they must be addressed to maximize the reproducibility of the data extracted.

## 5. Conclusions

The methods for measuring NAE in PMN and other leukocytes that release extracellular traps are contingent on the expertise of the researcher. The method presented here effectively mitigates the potential bias that may be introduced by researchers who are not yet fully trained and are not yet acquainted with the subtleties and pitfalls associated with the manual recognition of NAE. It is clear that the process has several limitations: It is time-consuming, requires managing large volumes of data, and requires access to TissueFAXS facilities. However, after addressing these limitations, we found that the method is reproducible and reliable. Therefore, this method is an effective tool for measuring early PMN activation. There are still some questions that will be answered in the future: is vital NETosis present in the early activation of PMN by spermatozoa in this species? This type of NETosis has not been described in reproduction, and, to date, there is no evidence of this kind of NETosis yet. The other question relates to whether there are some receptors in PMN that activate NETosis in the presence of spermatozoa, and if so, of what kind? These questions will generate new insights about the physiology of NETosis activated by spermatozoa in mammals in the near future.

## Figures and Tables

**Table 1:** Different representation of NETotic NAE for (**A**) canine PMN or (**B**) bovine PMN in the absence or presence of spermatozoa. * Indicates significant differences between groups with *p* < 0.05.

(A) Canine					
Cell types	Incubation time (min)	Mean area PMN (μm^2^)	Standard deviation	Confidence interval	*p* value
PMN	15	37.33	1.923	(33.55–42.10)	0.00061 *
PMN + sperm		47.40	2.670	(40.77–54.04)	
PMN	120	35.18	5.214	(22.23–48.14)	0.0058 *
PMN + sperm		38.99	9.216	(16.10–61.88)	
**(B) Bovine**					
Cell types	Incubation time (min)	% nuclei PMN > 80 μm^2^	Standard deviation	Confidence interval	*p* value
PMN	15	0.4250	0.1595	(0.1712–0.6788)	0.0200 *
PMN + sperm		4.005	1.350	(1.856–6.154)	
PMN	60	0.4725	0.1702	(0.2017–0.7433)	0.0251 *
PMN + sperm		4.865	2.359	(0.8242–8.906)	
PMN	120	0.7750	0.4691	(0.0286–1.521)	0.0039 *
PMN + sperm		5.640	2.1706	(2.177–9.103)	
PMN	240	0.6075	0.3705	(0.0179–1.197)	0.0680
PMN + sperm		4.5430	1.884	(1.545–7.540)	

## Data Availability

The raw data supporting the conclusions of this article will be made available by the authors on request.

## Abbreviations

The following abbreviations are used in this manuscript:

ARTs	Assisted reproductive techniques.
FI	Fluorescence intensity.
FRT	Female reproductive tract.
HBSS	Hank’s balanced salt solution.
MPO	Myeloperoxidase.
NAE	Nuclear area expansion.
NE	Neutrophil elastase.
NETs	Neutrophil extracellular traps.
NOX	NADPH oxidase.
PAD4	Peptidyl arginine deiminase type IV.
PBMCs	Peripheral blood mononuclear cells.
PMN	Polymorphonuclear neutrophils.
ROI	Region of interest.
ROS	Reactive oxygen species.
RT	Room temperature.
